# Some Lessons Learned About Diabetes and COVID-19 During the Early Stage of the Epidemic in Norway

**DOI:** 10.1177/1932296820929371

**Published:** 2020-05-26

**Authors:** Kåre I. Birkeland

**Affiliations:** 1 Department of Transplantation Medicine, Institute of Clinical Medicine, University of Oslo and Oslo University Hospital, Norway

**Keywords:** COVID-19, diabetes, information work, telemedicine

I have learned that events that at first seem banal and unimportant may suddenly change my own and thousands of people’s life. In November 2019, a citizen of Wuhan, China was infected with the SARS-CoV-2 virus that five months later has spread to >2 million citizens throughout the world.^1,2^ On February 24th a physician returned to work at our hospital’s Department of Ophthalmology after a skiing holiday in northern Italy. Two days later he had symptoms of a common cold, was diagnosed with COVID-19, and infected four colleague vitreous surgeons^3^ The Department was virtually closed down, our hospital with 20 000 employees went into a state of emergency and in a couple of days, Norway was virtually shut down.

I have learned that type 1 diabetes often creates more concern, distress, and anxiety to patients than I used to think. And particularly so during a pandemic like the present, when social distancing and home confinement change the way of living, allow ample time to bother and the COVID-19 itself has particular impact on people with diabetes. All patients that I have spoken to during these weeks have heard that diabetes is as a risk factor for potential serious complications to COVID-19.^4,5^

Our hospital’s diabetes outpatient clinic rapidly turned into a “virtual” clinic with telemedicine covering most of the consultations. We learned that this may be convenient for both patients and health care personal, but also that there are major limitations due to lack of information from physical examinations and nonverbal communication. Many patients did not wish to come to the hospital for blood sampling due to fear of being infected and important parameters for regular diabetes follow-up like glycated hemoglobin, microalbuminuria, and assessment of renal function could not be monitored. On the other hand, people wearing continuous glucose monitoring, that not previously had downloaded their reports to their home computer, started to do so and discovered a new tool of value to monitor their diabetes.

The Norwegian Diabetes Association played a major role in bringing out qualified and relevant information to persons with diabetes. The organization worked in close collaboration with its assigned medical experts and the Directorate of Health, and strengthened its usual information channels available on phone, chat, and email. On March 13, a “telephone hotline” was established, where diabetes experts answered phones from patients and their relatives. That resulted in a quadrupling of incoming telephones from 30-40 to 150/day and gave the opportunity to spread relevant information directly to those concerned. Most questions related to whether the increased risk of a serious course of COVID-19 apply to all with diabetes and if they could continue to live together with a husband or child that were exposed to the virus, for example, through their work or peers. Many patients experienced increased blood glucose levels and need for higher insulin doses due to less physical activity and changed dietary habits. The Diabetes Association in collaboration with its experts issued statements about children with diabetes (not at particular risk) and about the use of SGLT2 inhibitors (stopping rules if infected).

The COVID-19 pandemic have struck less hard in Norway than in many other countries. The reported incidence rate is about 120/100 000, similar to the other Scandinavian countries and in the “mid-European range.” However, the death rate per million population as of April 16 is only 24, which is far below the top European countries (>300), and lower than in our neighboring countries Sweden (119) and Denmark (53). One reason may be the unlucky ophthalmologist that spread the infection to his colleagues, and attained a lot of media attention that resulted in a brisk and comprehensive response from the government.

In the future, I predict that telemedicine will be more widely used in diabetes care, the information work to the public and the patients will be streamlined and reinforced on all media platforms, and the protective hygiene measures applied in general and at the outpatient clinics will be strengthened to avoid infections.

## References

[1] Zhu N Zhang D Wang W, et al A novel coronavirus from patients with pneumonia in China, 2019. N Engl J Med. 2020;382(8):727-733. 31978945 10.1056/NEJMoa2001017 PMC7092803

[2] Our World in Data. Total confirmed COVID-19 deaths per million people, 4 16, 2020 https://ourworldindata.org/grapher/total-covid-deaths-per-million?year=2020-04-16. Accessed April 16, 2020.

[3] Jørstad ØK Moe MC Eriksen K Petrovski G Bragadóttir R Coronavirus disease 2019 (COVID-19) outbreak at the Department of Ophthalmology, Oslo University Hospital, Norway. Acta Ophthalmol (Copenh). 2020;98(3):e388-e389. 10.1111/aos.14426 32227668

[4] Zhou F Yu T Du R, et al Clinical course and risk factors for mortality of adult inpatients with COVID-19 in Wuhan, China: a retrospective cohort study. Lancet. 2020;395(10229):1054-1062. 32171076 10.1016/S0140-6736(20)30566-3 PMC7270627

[5] Yang X Yu Y Xu J, et al Clinical course and outcomes of critically ill patients with SARS-CoV-2 pneumonia in Wuhan, China: a single-centered, retrospective, observational study. Lancet Respir Med. 2020;8(5):475-481. 32105632 10.1016/S2213-2600(20)30079-5 PMC7102538

